# Erratum for Rockey et al., “Seasonal influenza viruses decay more rapidly at intermediate humidity in droplets containing saliva compared to respiratory mucus”

**DOI:** 10.1128/aem.02508-25

**Published:** 2026-01-08

**Authors:** Nicole C. Rockey, Valerie Le Sage, Linsey C. Marr, Seema S. Lakdawala

## ERRATUM

Volume 90, no. 2, e02010-23, 2024, https://doi.org/10.1128/aem.02010-23. Page 6, Table 1: “Total protein, mg/mL” should read “Total protein, µg/mL.”

