# Supplementary material for: Seasonal influenza viruses decay more rapidly at intermediate humidity in droplets containing saliva compared to respiratory mucus
Source: Appl Environ Microbiol. 2024 Jan 9;90(2):e02010-23. doi: 10.1128/aem.02010-23 (PMC10880610; doi:10.1128/aem.02010-23)
Supplement: Supplemental figures — Figures S1 to S3. [file aem.02010-23-s0001.pdf]

Supplemental Material for:

Seasonal influenza viruses decay more rapidly at intermediate humidity in droplets containing saliva compared to respiratory mucus

Authors: Nicole C. Rockey,[1] Valerie Le Sage,[1] Linsey C. Marr,[2] Seema S. Lakdawala[1,3*]

1. Department of Microbiology & Molecular Genetics, The University of Pittsburgh, Pittsburgh, Pennsylvania, USA
2. Department of Civil and Environmental Engineering, Virginia Tech, Blacksburg, Virginia, USA
3. Department of Microbiology & Immunology, Emory University, Atlanta, Georgia, USA

*Corresponding author

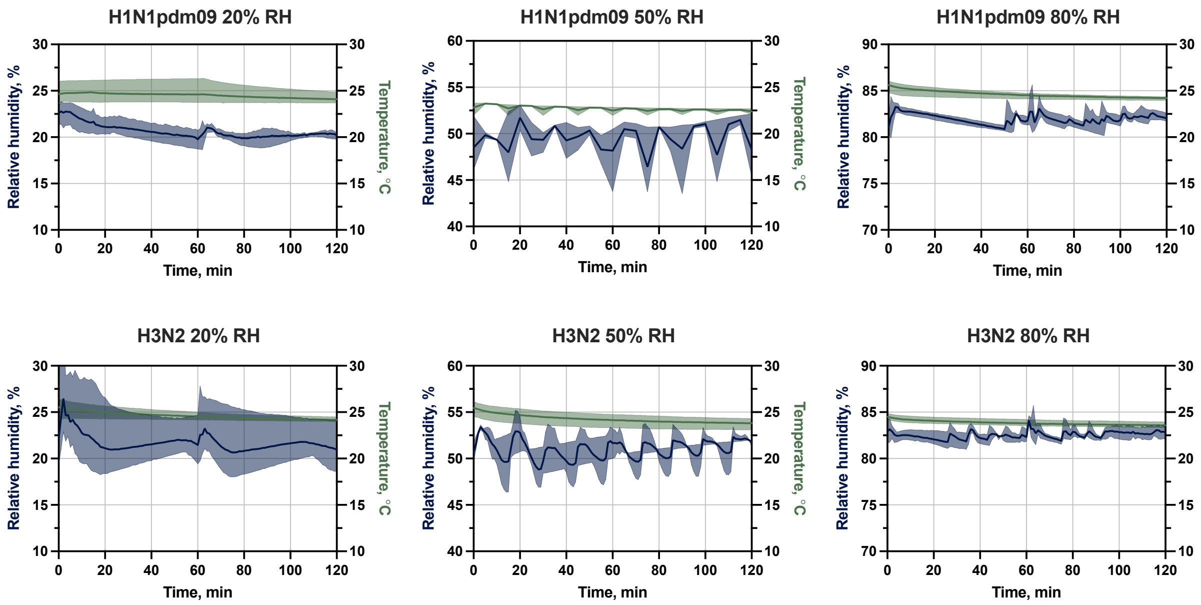

Figure S1. Relative humidity and temperature during droplet experiments. Mean relative humidity (blue) and temperature (green) of three independent replicates is indicated with a solid line for each condition tested using H1N1pdm09 or H3N2, and the shaded regions indicate the range of relative humidity and temperature.

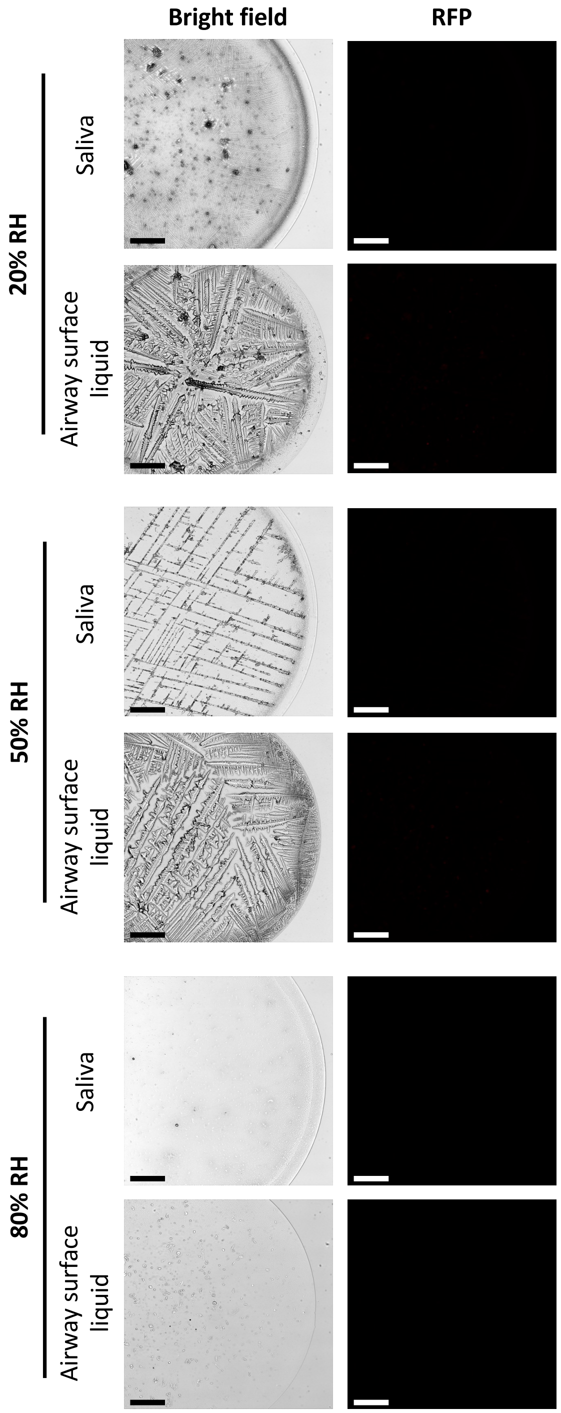

Figure S2. Negative fluorescence controls of droplets comprised of human saliva or airway surface liquid after drying for two hours at 20%, 50%, or 80% RH. Images were taken at 10x magnification. RFP = red fluorescence protein. Scale bars, 200 μm.

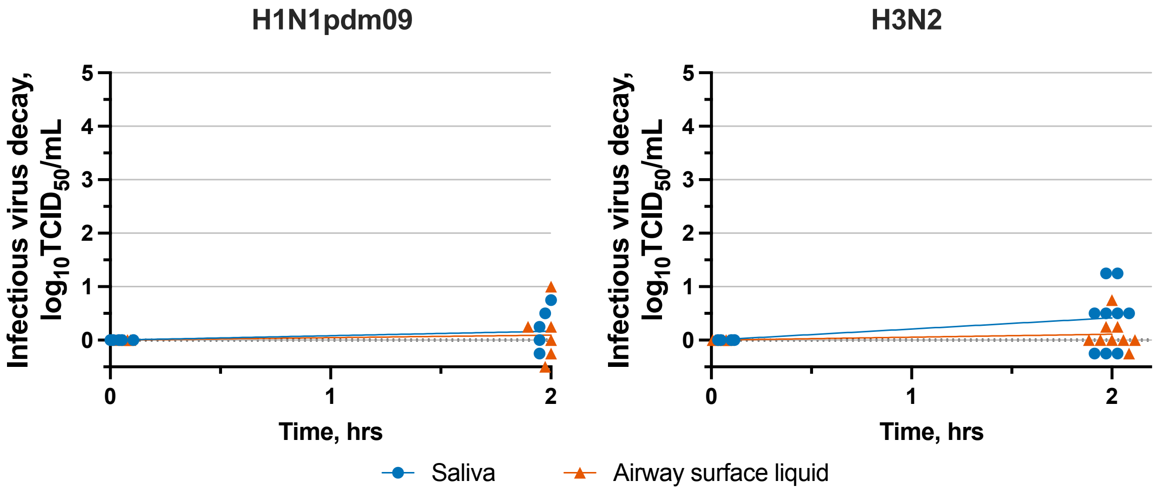

Figure S3. Bulk solution controls of saliva and airway surface liquid containing H1N1pdm09 or H3N2 do not show substantial decay. Samples of the infectious influenza virus in bulk solutions used for droplet generation were taken before and after the decay experiments conducted in Fig. 1 to assess any virus degradation in these respiratory fluids not associated with decay in drying droplets at variable RH. Bulk solutions were stored in microcentrifuge tubes in the biosafety cabinet over the course of each experiment (~ two hours) at ambient temperature. n = 8 and 9 for saliva and airway surface liquid, respectively.
